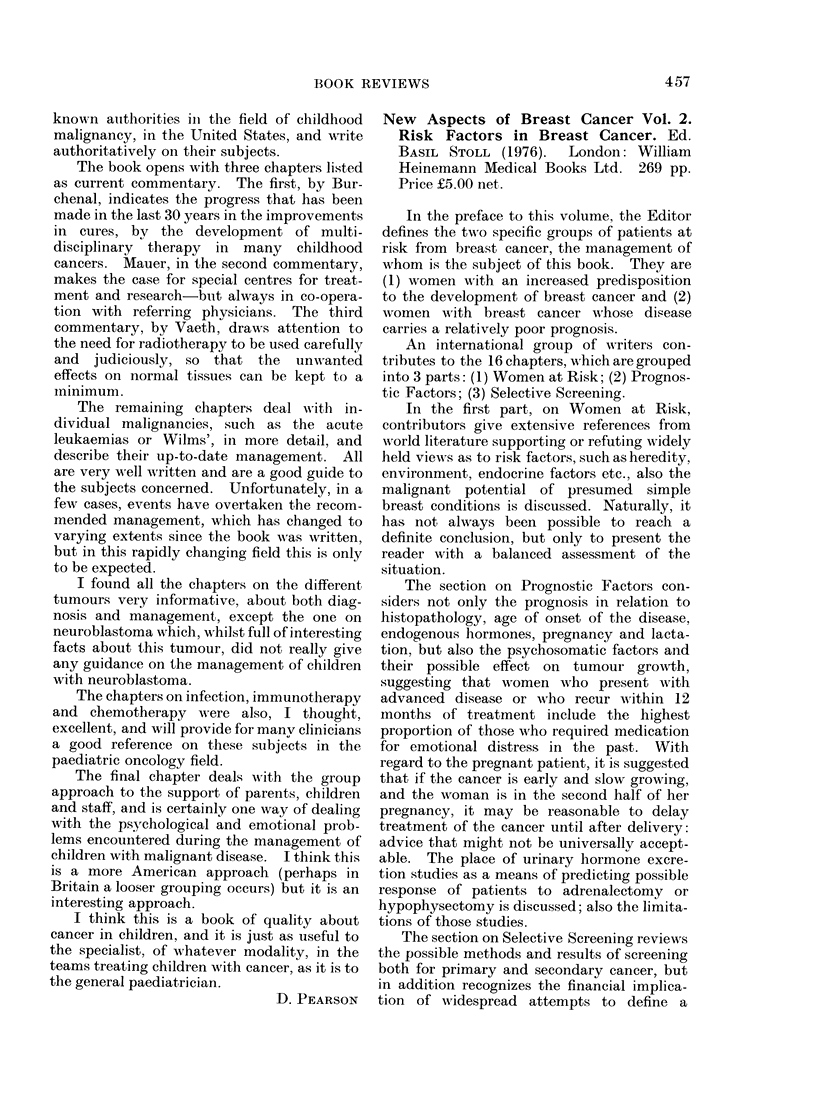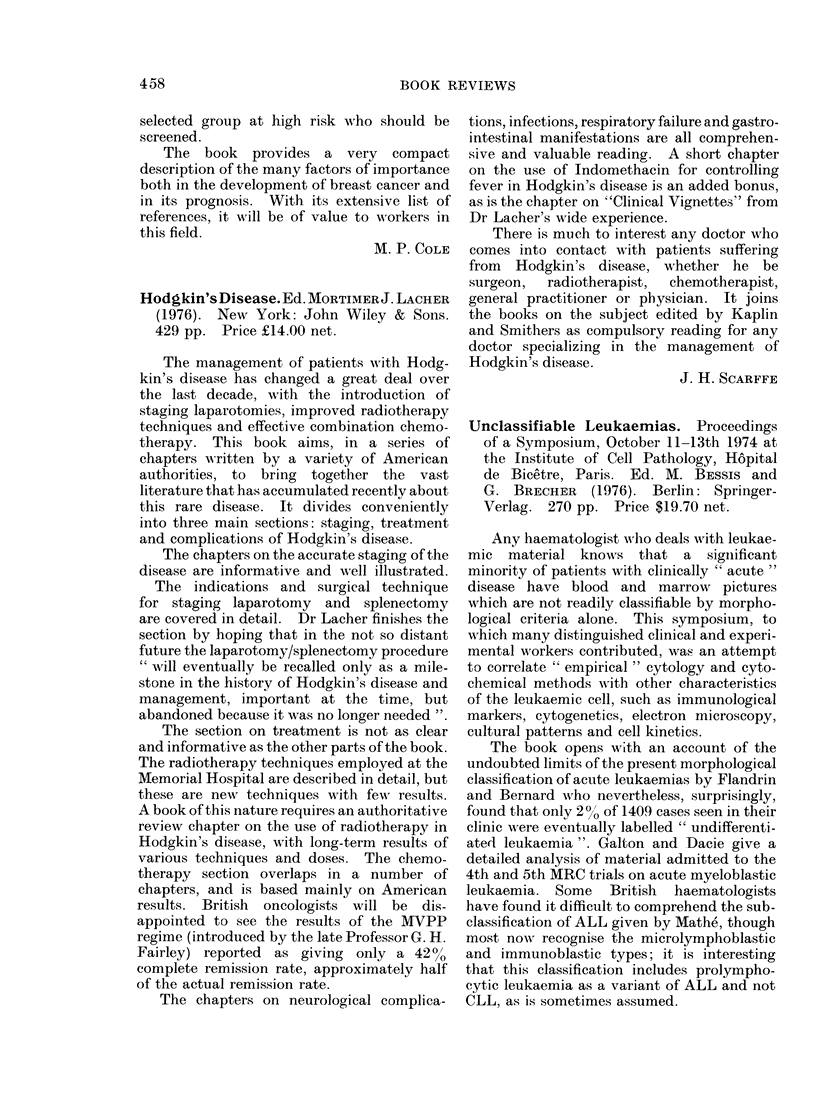# New Aspects of Breast Cancer Vol. 2. Risk Factors in Breast Cancer

**Published:** 1976-10

**Authors:** M. P. Cole


**New Aspects of Breast Cancer Vol. 2. Risk Factors in Breast Cancer.** Ed. BASIL STOLL (1976). London: William Heinemann Medical Books Ltd. 269 pp. Price £5.00 net.

In the preface to this volume, the Editor defines the two specific groups of patients at risk from breast cancer, the management of whom is the subject of this book. They are (1) women with an increased predisposition to the development of breast cancer and (2) women with breast cancer whose disease carries a relatively poor prognosis.

An international group of writers contributes to the 16 chapters, which are grouped into 3 parts: (1) Women at Risk; (2) Prognostic Factors; (3) Selective Screening.

In the first part, on Women at Risk, contributors give extensive references from world literature supporting or refuting widely held views as to risk factors, such as heredity, environment, endocrine factors etc., also the malignant potential of presumed simple breast conditions is discussed. Naturally, it has not always been possible to reach a definite conclusion, but only to present the reader with a balanced assessment of the situation.

The section on Prognostic Factors considers not only the prognosis in relation to histopathology, age of onset of the disease, endogenous hormones, pregnancy and lactation, but also the psychosomatic factors and their possible effect on tumour growth, suggesting that women who present with advanced disease or who recur within 12 months of treatment include the highest proportion of those who required medication for emotional distress in the past. With regard to the pregnant patient, it is suggested that if the cancer is early and slow growing, and the woman is in the second half of her pregnancy, it may be reasonable to delay treatment of the cancer until after delivery: advice that might not be universally acceptable. The place of urinary hormone excretion studies as a means of predicting possible response of patients to adrenalectomy or hypophysectomy is discussed; also the limitations of those studies.

The section on Selective Screening reviews the possible methods and results of screening both for primary and secondary cancer, but in addition recognizes the financial implication of widespread attempts to define a selected group at high risk who should be screened.

The book provides a very compact description of the many factors of importance both in the development of breast cancer and in its prognosis. With its extensive list of references, it will be of value to workers in this field.

M. P. COLE